# A Scoping Review of Viral Diseases in African Ungulates

**DOI:** 10.3390/vetsci8020017

**Published:** 2021-01-22

**Authors:** Hendrik Swanepoel, Jan Crafford, Melvyn Quan

**Affiliations:** 1 Vectors and Vector-Borne Diseases Research Programme, Department of Veterinary Tropical Disease, Faculty of Veterinary Science, University of Pretoria, Pretoria 0110, South Africa; h.end.rik@hotmail.com (H.S.); jannie.crafford@up.ac.za (J.C.); 2 Department of Biomedical Sciences, Institute of Tropical Medicine, 2000 Antwerp, Belgium

**Keywords:** foot and mouth disease, African swine fever, rift valley fever, bluetongue, lumpy skin disease, peste des petits ruminants, small ruminant morbillivirus, African horse sickness

## Abstract

(1) Background: Viral diseases are important as they can cause significant clinical disease in both wild and domestic animals, as well as in humans. They also make up a large proportion of emerging infectious diseases. (2) Methods: A scoping review of peer-reviewed publications was performed and based on the guidelines set out in the Preferred Reporting Items for Systematic Reviews and Meta-Analyses (PRISMA) extension for scoping reviews. (3) Results: The final set of publications consisted of 145 publications. Thirty-two viruses were identified in the publications and 50 African ungulates were reported/diagnosed with viral infections. Eighteen countries had viruses diagnosed in wild ungulates reported in the literature. (4) Conclusions: A comprehensive review identified several areas where little information was available and recommendations were made. It is recommended that governments and research institutions offer more funding to investigate and report viral diseases of greater clinical and zoonotic significance. A further recommendation is for appropriate One Health approaches to be adopted for investigating, controlling, managing and preventing diseases. Diseases which may threaten the conservation of certain wildlife species also require focused attention. In order to keep track of these diseases, it may be necessary to consider adding a “Wildlife disease and infection” category to the World Organisation for Animal Health-listed diseases.

## 1. Introduction

### 1.1. Rationale

Viral diseases are important as they can cause significant clinical disease in both wild and domestic animals, as well as in humans. Viral diseases make up a large proportion of emerging infectious diseases [1,2].

The past few decades have seen some diseases emerge, and re-emerge, that have had catastrophic effects on human and animal health. Emerging infectious diseases pose a significant threat to global public health and a large percentage (>60%) are zoonotic [2,3]. Emerging diseases have become more important because of growing populations of human beings and domestic animals, culminating in a surge in the emergence of zoonotic diseases [4,5]. Furthermore, some diseases which were once geographically isolated are now becoming global disease issues and threats due to the ease of travel and trade in animals and animal products [4,6]. Emerging diseases are of particular importance in developing countries as they can have a profound negative impact on food security and the livelihoods of poverty-stricken people. In addition, emerging diseases pose a major economic burden in both developing and developed countries as large amounts of money need to be spent in order to prevent disease emergence and maintain ongoing surveillance for emerging diseases [4,7].

Viral diseases are of particular importance in the African context as many of them affect more than one species of animal and pose a significant threat to entire ecosystems as biodiversity, animal behaviour and animal population composition can be affected. As a result, some species have even been pushed to the brink of extinction by several factors, including viral diseases [4,7]. The management and prevention of these diseases have proven to be challenging due to the large population of reservoir hosts consisting of African wildlife [1,8]. In South Africa, the wildlife industry forms a major part of both the agricultural and tourism sectors and contributes greatly to the country’s economy [9]. This industry suffers both direct (mortality and reduced productivity) and indirect losses (management and prevention costs, trade losses, reduced value of animals and food insecurity) due to infectious diseases [10].

Wildlife and the specific diseases infecting them are often neglected in studies and wild animals are rather categorised according to their epidemiological role as hosts, usually spillover, maintenance or dead-end hosts [2]. There have been a large number of studies investigating specific viral diseases and numerous diseases of significance have been identified [1,8].

There are several viruses known to cause clinical disease in African ungulates and a proportion of these viruses have been diagnosed only in captive-bred wildlife. The aim of this study was to identify those viruses which have been detected in free-ranging wildlife. The viral diseases known to be present in African wildlife include, but are not limited to, foot and mouth disease, rabies, African horse sickness, African swine fever, Rift Valley fever, bluetongue, lumpy skin disease, malignant catarrhal fever, encephalomyocarditis of elephants, peste des petits ruminants, canine distemper and feline immunodeficiency syndrome [1].

The pathogens which form the basis of this paper are viruses which have been isolated in African ungulates. This excludes domestic (e.g., sheep, cattle, goats and pigs) and feral ungulates (e.g., camels).

There is no comprehensive publication reviewing the publications on viruses in African ungulates. This research study aimed to fill this gap and provide comprehensive analyses to add to the current global knowledge base and provide guidance about areas lacking knowledge.

### 1.2. Aim

Perform a scoping review of viral diseases that occur in free-ranging African ungulates and identify knowledge gaps with regard to these diseases.

### 1.3. Objectives

1. Describe viruses diagnosed in free-ranging African ungulates.
2. Identify ungulates affected by viruses.
3. Describe the geographical distribution of viruses.
4. Identify viruses which appear to be “under-studied”.

### 1.4. Research Topic and Questions

The study consisted of a scoping review. At the start of the study, a team of experts in the fields of microbiology, research and data gathering was established. This team constructed the topic of the study as well as the study protocol, which included the databases to be searched and the development of search strings.

The population, interest and context (PICO) framework was modified and used to develop the research topic and questions. The population in focus was African ungulates, the interest was viral diseases of these animals and the context was to establish what the current global knowledge base is and to identify gaps in the knowledge base [10].

“Knowledge synthesis” denotes the integration of results obtained from individual research studies pertaining to a specific disease, topic or question into the global knowledge base [11]. A scoping review is the most suitable method of knowledge synthesis by which existing knowledge is mapped to areas in the global knowledge base where a lack of comprehensive analyses exists [12,13,14].

Knowledge synthesis methodologies were applied in this paper to deliver a comprehensive overview of published research on viral diseases of African ungulates. The intention was to quantitatively characterise peer-reviewed research with respect to the author, date of publication, reference type, animal species involved, virus involved, how the disease was diagnosed and temporal and regional patterns to establish the focus of research on viral diseases and to identify any gaps.

## 2. Materials and Methods

The methodology for this scoping review was based on the guidelines as set out in the Preferred Reporting Items for Systematic Reviews and Meta-Analyses (PRISMA) extension for scoping reviews. The PRISMA extension for scoping reviews was recently developed and provides standardised definitions and guidelines for scoping reviews [15]. Appendix A consists of the PRISMA checklist containing information relevant to this scoping review [15]. This study was conducted systematically in four main steps: firstly, the development of the research topic with relevant questions; secondly, the literature search was conducted by researching and identifying relevant publications; thirdly, screening and sorting of search results was conducted; and finally, data extraction and analyses were performed.

Ungulates were generally defined as animals possessing hooves and belonging to the orders *Perissodactyls* (odd-toed ungulates) and *Artiodactyls* (even-toed ungulates). Elephants (*Loxodonta africana*) were classified as ungulates for completeness as they are part of the clade *Paenungulata* (sub-ungulates). African was used to describe and define the ungulates as originating from Africa and refers to non-domestic ungulates and, hence, does not include indigenous domestic African cattle, sheep or goats, nor does it include feral ungulates found in Africa, e.g., camels. African ungulates that were described as being free-ranging or captive were included in the study and differentiated as such. For the purpose of this study, captive African ungulates were defined as ungulates that are indigenous to Africa and have been born and bred in captivity or have been captured with the purpose to be permanently captive animals—for example, animals held in zoological collections or intensively managed operations. Animals captured and held in a boma facility or smaller enclosures prior to relocation or transport were not classified as captive. Furthermore, free-ranging African ungulates were defined as ungulates that are indigenous to Africa and live free from direct human interaction and interventions for most of their lives. This includes animals in national and private game reserves and animals on game farms, as those in Southern Africa, which are managed extensively. Hence, wildlife was categorised as free-ranging as long as their management was deemed extensive.

### 2.1. Protocol

The protocol was approved by the Research Committee of the Faculty of Veterinary Science, University of Pretoria and can be provided upon request.

### 2.2. Eligibility Criteria

The eligibility criteria were set by three members of the research team. The screening process was initiated by three members of the research team but, due to time and geographical constraints, it was completed by one team member.

A two-stage screening process was implemented to evaluate the relevance of publications obtained during the search process.

### 2.3. Information Sources

Three major veterinary databases were used to obtain publications for this study, namely SciVerse Scopus (multidisciplinary, 1823–present), EBSCO Wildlife and Ecology Studies Worldwide (wildlife and ecology studies, 1892–present) and ISI Web of Science (multidisciplinary, 1900–present).

No language, date, subject or type filters were used during the searches, which allowed for a comprehensive search and reduced limitations on publications obtained. All databases were searched using the topic search function: this searched titles, abstracts and keywords of each publication and included publications from the databases’ inception to November 2019. Several publications were also obtained by performing a reverse reference search strategy on relevant references within obtained publications [16,17,18,19,20,21,22,23,24].

The initial search was performed in January 2019. A final follow-up search of the three scientific databases was performed in November 2019 to identify any new studies published that were relevant to viral diseases in African ungulates since January 2019.

### 2.4. Search

A base search string was developed using terms that were deemed relevant and descriptive of the publications required for inclusion in this scoping review. “Africa*” was used as the geographic search term to limit results to the African continent. No other geographical restrictions were applied. Viruses were not specifically searched for, but rather, a broad search term was developed to include any viruses or viral diseases; this was done in order to prevent the exclusion of any viruses not previously diagnosed in African ungulates. The search terms for viruses and viral diseases were “virus OR viral”. Search terms for the indigenous African ungulates were based on the common genus names as well as the Latin genus or species names.

This base search string was adapted to meet the requirements of the individual database search engines; Appendix B contains the complete search strings. The base search string was as follows:

Africa* AND (virus OR viral) AND (loxodonta OR “african elephant” OR giraff* OR syncerus OR “african buffalo” OR “cape buffalo” OR hippopotamus OR choeropsis OR rhinoceros OR ceratotherium OR diceros OR “equus zebra” OR “equus africanus” OR grevyi OR quagga OR phacochoerus OR warthog OR potamochoerus OR bushpig OR “red river hog” OR aepyceros OR impala OR alcelaphus OR hartebees* OR connochaetes OR wildebees* OR damaliscus OR tsessebe OR bonteb* OR blesb* OR antidorcas OR springb* OR raphicerus OR steenb* OR grysbok OR tragelaphus OR kudu OR koedoe OR nyala OR bongo OR bushbuck OR bosbok OR sitatunga OR taurotragus OR eland OR hippotragus OR sable OR roan OR oryx OR gemsb* OR pelea OR rheb* OR redunca OR reedbuck OR rietbok OR “kobus ellipsiprymnus” OR waterb* OR “kobus leche” OR lechwe OR “kobus kob” OR “kobus vardonii” OR puku OR cephalophus OR sylvicapra OR philantomba OR duiker OR oreotragus OR klipspringer OR spekei OR leptoceros OR “Gazella dorcas” OR eudorcas OR nanger OR addax OR “capra nubiana” OR “nubian ibex” OR beatragus OR hirola OR ammotragus OR “barbary sheep” OR dorcatragus OR madoqua OR “dik-dik” OR okapia OR okapi OR neotragus OR “royal antelope” OR suni OR litocranius OR gerenuk OR hyemoschus OR chevrotain OR ourebia OR oribi) AND NOT (beetle OR arthropod OR oryctes OR nudivirus OR javan OR sumatran OR “one horned” OR snake OR chicken* OR human* OR Newcastle OR arabian OR tragus OR aquaculture OR waterborne).

#### 2.4.1. Citation Management

All publications obtained during the search process were imported into EndNote X8 (Clarivate Analytics, Philadelphia, PA, USA). Duplicate publications were removed via EndNote’s automated duplicate screening process and several more duplicates were removed manually where minor differences in the title (e.g., using uppercase letters instead of lowercase letters) did not allow EndNote to detect the duplicate. All publications underwent manual title and abstract screening for relevance and then full-text screening using EndNote X8 software.

#### 2.4.2. Inclusion and Exclusion Criteria

Publications were eligible to be included in the study if the full-text article was written in English and if they described general or specific viruses or viral diseases in any African ungulates. Publications that reported on viruses or viral diseases in African ungulates in zoos/captivity, experimental studies or viruses/viral diseases in vectors were handled separately. A publication was excluded if it involved viruses or viral disease in domestic species, primates, rodents, bats or invasive species, or if it was a review paper.

### 2.5. Selection of Sources of Evidence

#### 2.5.1. Title and Abstract Relevance Screening

The first screening level involved review of only the title and the abstract of publications. Publications without keywords referring to Africa or African countries, viruses or viral diseases and any of the African ungulates in their title, abstract and keywords were excluded. Irrelevant publications were obtained due to search terms having similar meanings, different truncation rules, different search algorithms and other database settings which the user did not have control over. This allowed a large proportion of non-relevant publications to be identified and excluded, saving time that would have been spent procuring the full text and performing full-text screening of the excluded publications.

#### 2.5.2. Full-Text Screening

The full texts of relevant publications identified by the title and abstract screening were obtained via several methods. Some were obtained using the full-text procurement function of EndNote that was linked to the library service of the University of Pretoria (UP). The majority were obtained by searching for the title in Google Scholar that was also linked to the UP Library service and directly via the UP Library services’ database search function. A small number of publications required procurement by request from other university libraries; this was orchestrated by one of the team members who had expertise in research and data gathering. Some publications obtained from international university libraries were unobtainable in English and hence were excluded based on language.

The full-text articles were screened for eligibility and, if criteria were not met, the publications were excluded at this step. Once the final set of full-text publications was constructed, data were extracted from the publications.

### 2.6. Data Charting Process

Data extraction and charting were performed using EndNote X8 software and Microsoft Access Office 365 (Microsoft Corporation, Redmond, DC, USA). Tables were created in the database for the data items detailed below. A data charting form was used to capture information relevant to answering the research questions and objectives. The data charting form is available in Appendix C.

### 2.7. Data Items

Specific data extracted from relevant studies were as follows:

1. Reference
  - EndNote reference number, first author surname and date of publication
2. Reference type
  - Assay (Antibody) development
  - Assay (Antigen) development
  - Assay (molecular) development
  - Case/outbreak report
  - Phylogenetic study
  - Surveillance
  - Experiment
3. Animal
  - Genus
  - Species
4. Range
  - Free-range
  - Captive
5. Virus
  - Family
  - Genus
  - Species
6. Diagnosis
  - Clinical signs (positive diagnosis)
  - Laboratory—viral isolation (positive diagnosis)
  - Laboratory—antigen detection (positive diagnosis)
  - Laboratory—molecular detection (positive diagnosis)
  - Laboratory—antibody detection (positive diagnosis)
  - Clinical signs (negative diagnosis)
  - Laboratory—viral isolation (negative diagnosis)
  - Laboratory—antigen detection (negative diagnosis)
  - Laboratory—molecular detection (negative diagnosis)
  - Laboratory—antibody detection (negative diagnosis)
7. Outbreak
  - Year of data collected, study performed, publication or outbreak/case report
8. Country
  - Includes all African countries
9. Latitude (of outbreak)
10. Longitude (of outbreak)
11. Quantitative data (yes/no)
12. Comments

### 2.8. Classification of High-Impact Viruses

High-impact viruses are generally defined as viruses that have a significant negative impact on the health and lives of animals and humans due to their high morbidity/mortality rates in livestock, negative economic impacts and zoonotic potential [10,25]. For the purposes of this scoping review, a list of high-impact viruses was derived from the list of notifiable diseases by the World Organisation for Animal Health (OIE) [26].

### 2.9. Critical Appraisal of Individual Sources of Evidence

A critical appraisal of each publication did not take place prior to data extraction due to time constraints. However, all publications are peer-reviewed quantitative and/or qualitative research.

### 2.10. Synthesis of Results

The Microsoft Access Office 365 database allowed the construction of queries to calculate descriptive and quantitative results to summarise the data. Results were depicted as maps, graphs and plots using Microsoft Excel Office 365 (Microsoft Corporation, Redmond, DC, USA) and ArcGIS Desktop 10.6 (Environmental Systems Research Institute, Redlands, DC, USA).

### 2.11. Presentation of Results

Results were presented in a quantitative format. In some instances, this may imply that a virus with the highest number of detections in several host species is the most important one. However, in reality, this may not be the case.

## 3. Results

### 3.1. Selection of Sources of Evidence

The number of publications retrieved from each database for each of the two searches were as follows:

- Scopus
  ○ January 2019—248 publications, removed duplicates and 237 left
  ○ November 2019—11 new publications, all irrelevant
- Wildlife and Ecology Studies Worldwide
  ○ January 2019—79 publications, removed duplicates and 45 left
  ○ November 2019—1 new publication, duplicate and irrelevant
- Web of Science
  ○ January 2019—48 publications, removed duplicates and 44 left
  ○ November 2019—1 new publication, duplicate and irrelevant

The initial search performed during January 2019 returned 375 potentially relevant publications. Following duplicate removal, 326 publications remained and progressed to the title and abstract screening stage. Following screening for relevance based on title and abstract, 160 remained and entered the full-text screening process. The full-text articles for these publications were obtained for review. During the full-text screening process, 11 publications were identified and obtained via a reverse reference search and added to the cohort of publications to be screened. Nine of these “reverse reference searched” publications remained following title and abstract screening. Thus, 169 publications entered the full-text screening process. Seven full-text articles could not be obtained, three were not available in English (two were in French and one in German), seven did not meet the inclusion criteria, two had duplicate results (same data in two publications) and five discussed *African swine fever virus* isolation from ticks but not wild suids; hence, these 24 publications were excluded from this scoping review.

A follow-up search was performed during November 2019 and returned 13 potentially relevant publications. Following duplicate removal and screening for relevance based on title and abstract, 0 publications remained.

No filters were set for any of the searches and publications were only screened for language requirements once they reached the full-text screening phase.

**Figure 1:**
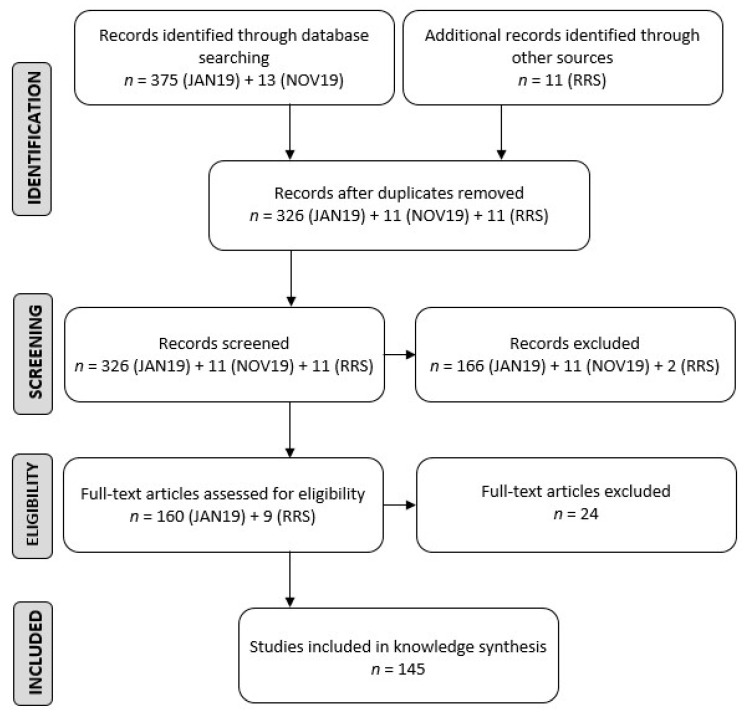
Flow diagram indicating the number of publications reviewed and excluded during each step of the review process, RRS—Reverse Reference Search.

One hundred and forty-five publications made up the final set of publications included in the scoping review. Figure 1 indicates the number of publications reviewed and excluded during each step of the review process [27]. It does not reflect the chronological order of events but rather the total number of publications included in each step of the review process. Despite including only 145 publications, some publications consisted of more than one study type, some mentioned more than one virus or viral disease and some mentioned more than one animal species; hence, the total reports of viral diseases in African ungulates for the different categories amounted to greater than 145.

### 3.2. Synthesis of Results

#### 3.2.1. General Characteristics of Reported Publications

**Figure 2:**
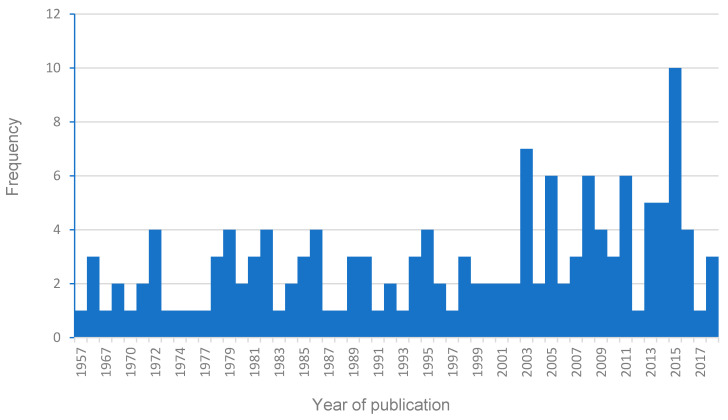
Number of publications reporting viral diseases in African ungulates per year from 1957 to 2018.

The range of the publication dates was from 1957 to 2018 (Figure 2). Sixteen percent (23/145) of publications were published from January 2014 to December 2018 and 50% (72/145) of studies were published between the start of 2001 and the end of 2018. The highest number of publications (10) in a year was in 2015.

Most publications were surveillance studies, constituting 40% (58/145) of the total publications (Table 1). The total number of publications in Table 1 was 148 even though the total number of publications was only 145. The reason for this discrepancy was that some publications contained more than one study type; for example, both an experiment and surveillance study and counted more than once in the database. Furthermore, a large majority of the publications (95%) (138/145) reported on viruses in free-ranging African ungulates and only 5% (7/145) of publications reported on viruses in captive African ungulates.

#### 3.2.2. Viruses Reported and Diagnosed in African Ungulates

A total of 32 viruses were reported by the 145 publications in African ungulates (Table 2). The five viruses with the most publications reporting on them in African ungulates, in descending order, were *Foot and mouth disease virus* (32% of publications, 46/145), *African swine fever virus* (14% of publications, 20/145), *Alcelaphine gammaherpesvirus 1* (12% of publications, 17/145), *Rift Valley fever phlebovirus* (6% of publications, 9/145) and *Elephantid betaherpesvirus 1/4/5* (6% of publications, 8/145). The remaining 27 viruses only had 30% of publications report on them.

The total number for publications in Table 2 was 173, despite only 145 publications being included in this research study. The reason for this discrepancy is that some publications reported on more than one virus/viral disease in African ungulate species and counted more than once in the database.

The number of reports that detected viral antigen/antibodies in African ungulates is shown in Table 3. *Foot and mouth disease virus* was detected the most frequently in publications, accounting for 20% (94/466) of the total reports of viruses detected, followed by *Bovine alphaherpesvirus 2* (11%—49/466), *Alcelaphine gammaherpesvirus 1* (9%—37/466), *Pestivirus A/B* (7%—32/466), *Bluetongue virus* (6%—28/466), *Bovine alphaherpesvirus 1* (6%—28/466) and *Bovine respirovirus 3* (6%—26/466), *African swine fever virus* (5%—24/466), *Rift Valley fever phlebovirus* (4%—20/466). These nine viruses alone accounted for 74% of the total reports of viruses detected by antigen/antibody testing in African ungulates.

**Figure 3:**
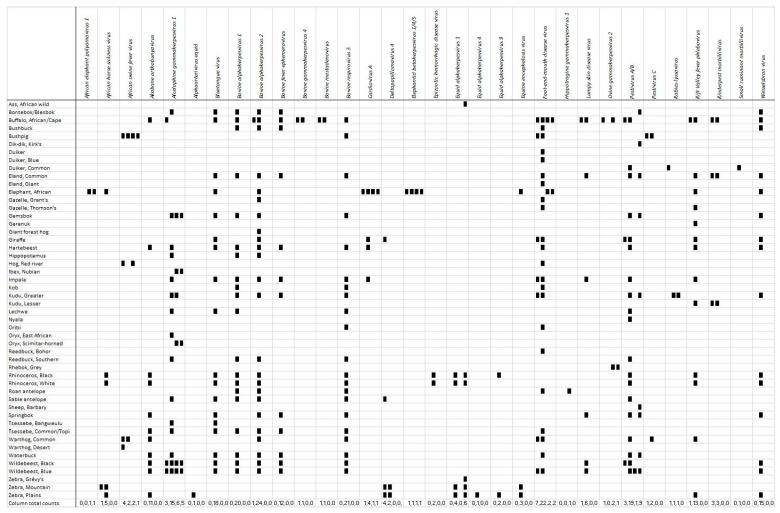
Detection of viral antigen/antibodies in free-ranging and captive African ungulates. The ungulate species are listed in the left-hand column and the pathogens (viruses) are listed in the first row. A black rectangle in the furthest left position within the cell indicates that the pathogen has been detected in a free-ranging member of that ungulate species. For example, *Alcelaphine gammaherpesvirus 1* antigen has only been detected in free-ranging African buffalo. A black rectangle in the centre left position within the cell indicates that antibodies to the pathogen have been detected in a free-ranging member of that ungulate species. For example, *Alcelaphine gammaherpesvirus 1* antibodies have only been detected in free-ranging Blesbok/Bontebok. A black rectangle in the centre right position within the cell indicates that antibodies to the pathogen have been detected in a captive member of that ungulate species. A black rectangle in the furthest right position within the cell indicates that the pathogen has been detected in a captive member of that ungulate species. For example, *African elephant polyomavirus 1* antigen and antibodies have been detected only in captive African elephants. These are not quantitative data about pathogens or antibodies to pathogens detected in ungulates but rather indicate whether a particular pathogen or antibodies to the pathogen have been detected in a specific ungulate species or not.

These reports were further classified according to the detection of viral antigen/antibody detected either in free-ranging or captive African ungulates (Figure 3). It is worth noting that *African elephant polyomavirus 1* and *Hippotragine gammaherpesvirus 1* have been detected only in captive animals according to the published literature using antigen/antibody detection as indicated by the zero total counts in the first two positions for each virus. The remainder of the viruses have either been diagnosed in a combination of free-ranging and captive animals, e.g., *Foot and mouth disease virus*, *African swine fever virus*, or only in free-ranging ungulates, e.g., *Akabane orthobunyavirus*, *Bluetongue virus*. It can be confirmed, by examining Figure 3, that the majority of viruses and/or viral antibodies have been detected in free-ranging ungulate species because most of the viruses in Figure 3 have counts for detections in free-ranging ungulates in the first two positions within the cells.

#### 3.2.3. Specific Ungulates Affected by Viruses

A wide variety of African ungulates were affected by viruses and a complete list is provided (Table 4). Of the 50 ungulate species affected by viruses, the four African ungulates with the most viruses diagnosed via antigen/antibody detection, in descending order, were the African buffalo, blue wildebeest, impala and warthogs (Table 3). African buffalo accounted for 17% (79/466) of antigen/antibody-diagnosed viruses in African ungulates. This was by far the ungulate with the most reports. Blue wildebeest accounted for 7% (34/466) of diagnosed viruses in African ungulates, followed by impala (6%—29/466) and warthogs (6%—27/466). The specific viruses with the most reports of being detected by antigen/antibody tests in specific ungulates were represented by 41 reports (8.8% of 466 reports) and 14 reports (3% of 466 reports) of *Foot and mouth disease virus* in African buffalo and impala, respectively. There were 16 reports (3% of 466 reports) of *African swine fever virus* in warthogs. There were also 12 reports (3% of 466 reports) of *Alcelaphine gammaherpesvirus 1* in blue wildebeest.

#### 3.2.4. Geographical Distribution of Viruses

Of the 54 countries on the African continent, only 18 (33%) had viruses diagnosed in free-ranging ungulates in the literature (Table 5).

**Figure 4:**
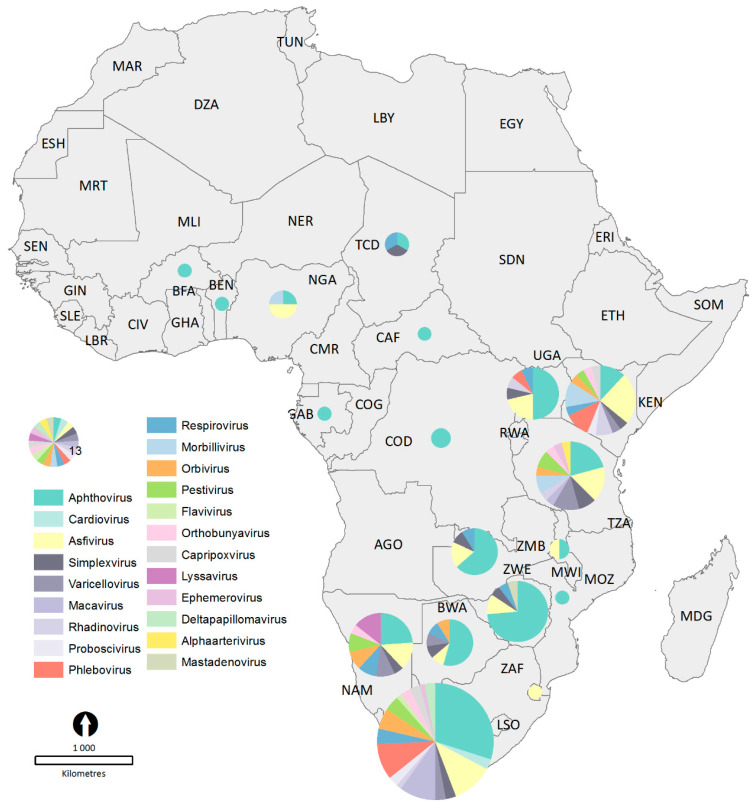
Geographical distribution of publications reporting on viruses in African ungulates.

Figure 4 provides a graphical depiction of the viruses reported in each country. Most reports of viruses originated from Southern Africa (South Africa, Namibia, Botswana, Zimbabwe, Zambia, Eswatini and Mozambique) and Eastern Africa (Tanzania, Kenya and Uganda) and a small proportion originated from Northern, Central and Western Africa. This confirms that all the publications in this study reported on viruses/viral diseases in ungulates from sub-Saharan Africa.

The total number in Table 5 was 240 despite Table 3 indicating 466. The reason for this discrepancy is that individual ungulate species were not considered for the reports detecting viral antigen/antibodies in each country—the report was counted based on the virus detected.

#### 3.2.5. Viruses which Seem to Be “Under-Studied”

Several of the 32 viruses reported in African ungulates are classified as high-impact viruses for the purposes of this study. The high-impact diseases which form part of the 32 reported diseases are:

- Foot and mouth disease
- African swine fever
- Rift Valley fever
- Bluetongue
- Rabies
- Lumpy skin disease
- Peste des petits ruminants
- African horse sickness
- Epizootic haemorrhagic disease
- Bovine viral diarrhoea (*Pestivirus A/B*)
- Infectious bovine rhinotracheitis/infectious pustular vulvovaginitis (*Bovine alphaherpesvirus 1*)
- Equine influenza (*Influenza A virus*)
- Equine viral arteritis (*Alphaarterivirus equid*)
- Equine viral rhinopneumonitis (*Equid alhpaherpesvirus 1*)
- Classical swine fever (*Pestivirus C*)

Foot and mouth disease, African swine fever, Rift Valley fever, bluetongue and rabies are frequently reported on in the literature. On the contrary, lumpy skin disease, peste des petits ruminants, African horse sickness, epizootic haemorrhagic disease, bovine viral diarrhoea, infectious bovine rhinotracheitis/infectious pustular vulvovaginitis, equine influenza, equine viral arteritis, equine viral rhinopneumonitis and classical swine fever are infrequently reported on (Table 3).

A breakdown of the number of African ungulate species affected by a virus family/genus/species is provided (Table 6). The five virus species that affected the widest ranges of African ungulates are, in descending order, *Bovine alphaherpesvirus 2* (24 of 50 ungulate species), *Foot and mouth disease virus* (23 of 50 ungulate species), *Pestivirus A/B* (22 of 50 ungulate species), *Bovine respirovirus 3* (21 of 50 ungulate species) and *Bovine alphaherpesvirus 1* (20 of 50 ungulate species).

## 4. Discussion

### 4.1. Summary of Evidence

This study provided a scoping review of the published literature on viruses and their associated diseases in African ungulates. To our knowledge, it is the first of its kind on this topic, with the scientific community showing increased interest in this area.

Several recommendations are outlined below for future research opportunities based on the general characteristics of reported publications, viruses reported and diagnosed in African ungulates, specific ungulates affected by viruses, the geographical distribution of viruses and viruses that seem to be “under-studied”. The intention of this scoping review was to provide a foundation for more focused analyses to be performed in future research projects. This will allow current knowledge to be built upon and new knowledge bases to be developed.

The search for publications to be included in this study was constructed so that it would be comprehensive but still practical as well as making efficient use of human and time resources. Publications reporting cases of viral disease or detection of viral antigens/antibodies or molecular viral isolation in African ungulates were relevant to this study. Consideration of the accuracy of the diagnosis made in the relevant publications broadly referring to viral disease in African ungulates was beyond the scope of this research study.

A number of publications were not detected during the search process by interrogating the database using the search string and were found via a reverse reference search process. The reason for publications not being detected during the search process was most likely due to the manner in which the databases’ search algorithms work. For example, the publications not initially detected may not have had specific words in their titles, abstracts or keyword lists or the correct combination of words between the three categories for the database algorithm to include the publications in the search results.

#### 4.1.1. General Characteristics of Reported Publications

Results show that around half of the publications that focused on viral diseases in African ungulates occurred during the eighteen years from the start of 2001 and the end of 2018. This confirmed that there is increasing interest in this field amongst scientists. The majority of publications (40%) were classified as surveillance studies, indicating that disease surveillance in African ungulates is a fairly common practice and data from these studies are readily published. This is likely due to the ease of performing surveillance for multiple diseases by simply collecting blood samples. Furthermore, a large majority of the publications discussed viruses in free-ranging African ungulates and only 5% of the publications discussed viruses in captive African ungulates. This is interesting as it would be expected that it would be easier to obtain samples from captive animals; however, the population sizes of captive African ungulates are small in comparison to their free-ranging counterparts and, prior to animals becoming captive, they are likely to undergo testing for certain diseases. If they provide a positive result, they are unlikely to be placed into a captive collection. In addition, animals that form part of zoological collections are tested for a large number of diseases to meet import/export conditions and for private testing schemes and sometimes these test results may not be reported. However, it is a requirement by the OIE to report any detections of OIE listed diseases in any species of animal [26].

#### 4.1.2. Viruses Reported and Diagnosed in African Ungulates

Based on the results, it has been established that many viruses can infect and, in some cases, cause disease in African ungulates. In addition, many of these viruses infect and cause disease in livestock too [28] and it has been shown that the exposure of wildlife to domestic animals and/or human-generated activities, such as deforestation, urbanisation and agricultural intensification, play a major role as drivers for the emergence of wildlife diseases [29].

The viruses of significance, according to the number of publications that have reported on them, are *Foot and mouth disease virus*, *African swine fever virus*, *Alcelaphine gammaherpesvirus 1* and *Rift Valley fever phlebovirus* (Table 2). These four viruses account for more than 50% (92/145) of the published research and reports on viral diseases in African ungulates. Based on the number of reports of viral antibody/antigen detected in African ungulates, *Foot and mouth disease virus*, *Bovine alphaherpesvirus 2*, *Alcelaphine gammaherpesvirus 1*, *Pestivirus A/B*, *Bluetongue virus*, *Bovine alphaherpesvirus 1*, *Bovine respirovirus 3*, *African swine fever virus* and *Rift Valley fever phlebovirus* featured amongst the viruses most detected in African ungulates (Table 3 and Figure 3). This is likely because several of the publications involved viral antigen/antibody surveillance of large numbers of wild African ungulates.

Foot and mouth disease and African swine fever are two of the diseases of high interest due to their economic importance but neither are zoonotic [30,31]. Zoonotic viral diseases, such as Rift Valley fever and rabies, are of high importance because of the disease they cause in humans [32,33]. These diseases of high interest generally stimulate public and political interest and will automatically attract funding for research. In comparison, some viral diseases exclusive to animals which are listed as being diseases of high impact, e.g., African horse sickness and peste des petits ruminants, have significantly less research associated with them, likely due to the fact that they are of low economic, political and zoonotic interest within the context of African wildlife [34,35]. In addition, most of the other diseases which have high numbers of reports of being detected by antigen/antibody testing in African ungulates do not cause serious clinical disease in free-ranging wildlife, at least not that has been documented [30,33,36,37].

*Foot and mouth disease virus* has the largest number of publications reporting on it and has been detected the most by antigen/antibody testing in African ungulates compared to the other 31 viruses (Table 3 and Figure 3). It is a virus of significance based on research and its impact on the global economy, but it does not cause clinically significant disease in free-ranging African ungulates [31]. It will only cause significant morbidity on occasion, specifically when the animals are stressed—for example, when animals are held in a boma facility for research purposes or relocation. A likely reason for foot and mouth disease receiving so much attention is that it is a highly trade-sensitive disease. This reflects the fact that funding into disease research is often driven by economic and political agendas [31,38]. In contrast, a virus such as rabies has a significantly smaller number of publications reporting on it in African ungulates despite causing widespread mortality and significant clinical disease, even in ungulates. Additionally, rabies is one of the most notable zoonotic diseases present and carries a significant threat to the health and conservation of wild carnivores [32,39,40].

With African swine fever, years of funding and research have provided very limited effectiveness in reducing outbreaks of the disease and, at the time of writing, there were major outbreaks occurring across Europe and Asia, initiated and driven by increased and easier global travel, trade in pork (legal and illegal) and poor biosecurity measures, e.g., feeding of animal products to animals [41]. Recently, there has been a change in research focus from wild suids to argasid ticks that are commonly found in warthog burrows and are responsible for the maintenance of *African swine fever virus* and socioeconomic factors that drive the spread of the disease. This indicates that scientists are realising that these are the key issues requiring attention, rather than wild suids being the reservoir of African swine fever. For most viruses, the impact of infection, whether they cause clinical or subclinical infection in African ungulates, is limited [42]. Transmission of African swine fever to domestic pigs at the wildlife/livestock interface in Africa is often suggested but a true interface is rarely documented [41]. The spread of African swine fever to Europe and Asia was driven by trade in pork and poor biosecurity measures with minimal involvement of wild suids. However, African swine fever has now become well established in the wild boar population in parts of Europe, resulting in its spread across Europe [41].

*Alcelaphine gammaherpesvirus 1*, causing malignant catarrhal fever, featured highly on the list of viruses when it came to the number of publications reporting on it and reports of its detection by antigen/antibody testing in African ungulates (Table 3 and Figure 3). This is interesting because, to date, very few clinical cases of malignant catarrhal fever have been reported in free-ranging African ungulates and a few cases have been reported in captive African buffalo [43]. The reason for this finding is most likely due to the fact that malignant catarrhal fever is readily transmitted from blue and black wildebeest to cattle in conditions where they live in close proximity to each other [43,44]. This confirms that certain viruses are not of great significance in African wildlife but are of significance to livestock producers and hence will receive funding and interest from the agricultural sector. In addition, given that the only free-ranging African ungulate in which clinical disease of malignant catarrhal fever has been reported is the African buffalo, it is recommended that malignant catarrhal fever surveillance and research take place in buffalo in the future as it may be an emerging viral disease in this species or a reservoir species of *Alcelaphine gammaherpesvirus 1* may be identified from which spillover occurs to the buffalo [43].

*Rift Valley fever phlebovirus* is a significant virus in the context of human, livestock and wildlife health; hence, it deserves to be listed as one of the viruses which had a high number of publications reporting on it and had a large number of reports of being detected by antigen/antibody testing in African ungulates (Table 3 and Figure 3). As an example, in 2010, there was an outbreak of Rift Valley fever in South Africa, with the first case being reported in January 2010 in the Free State province. By the end of the outbreak, the disease had been reported in eight of the nine provinces, KwaZulu-Natal being the only unaffected province [45,46]. It was also the first time in the history of Rift Valley fever outbreaks in South Africa that a winter rainfall area, i.e., the Western Cape, was affected [46]. The government reported 237 confirmed human cases of Rift Valley fever, with 26 deaths and large numbers of animals affected, including sheep, goats, cattle and wildlife [46,47]. Based on this outbreak, *Rift Valley fever phlebovirus* is evidently a pathogen of animal origin that has extended its host range and is able to infect humans. The outbreak seemed to be driven by climatic and ecological changes resulting in increased rainfall, as well as anthropogenic ecological changes (manmade dams and agricultural intensification) resulting in increased populations of mosquitoes. Despite ongoing research and the availability of vaccinations, this zoonotic disease, endemic to Africa’s tropical regions, is of significance as it has the potential to become a global emerging disease if a One Health management strategy is not implemented to manage it [48,49].

A noteworthy observation is that *Foot and mouth disease virus*, *Bovine alphaherpesvirus 2*, *Alcelaphine gammaherpesvirus 1*, *Pestivirus A/B*, *Bluetongue virus*, *Bovine alphaherpesvirus 1*, *Bovine respirovirus 3*, *African swine fever virus* and *Rift Valley fever phlebovirus* are all viruses of great significance in domestic livestock agriculture, hence the reason for their surveillance in wildlife, but only a few of them cause significant clinical disease in free-ranging African ungulates. Additionally, there was some overlap but also some discrepancy between the number of publications reporting on viruses and the reports of viral antigen/antibody detected in African ungulates, i.e., *African swine fever virus* and *Rift Valley fever phlebovirus* were lower on the ranking of viruses reported to be detected by antigen/antibody testing in African ungulates compared to the high ranking of viruses reported on by number of publications. A possible reason for this discrepancy with *African swine fever virus* may be that the virus is very hard to detect in wild suids and a large number of publications focused on its detection in argasid ticks, which were outside the scope of this research study. Surveillance of *African swine fever virus* in wild suids is very limited, resulting in a low ranking of viruses detected by viral antibody/antigen detection. In the case of *Rift Valley fever phlebovirus*, the virus has a narrow region of infection, generally tropical areas with high rainfall, and has recently become an emerging disease and spread to new geographical areas. Surveillance for *Rift Valley fever phlebovirus* has not been as significant as for some of the older viruses because it ranked lower on the list of viruses detected by viral antibody/antigen detection (Table 3).

*African elephant polyomavirus 1* is one of two viruses that was solely diagnosed in captive animals according to the published literature using antigen/antibody detection (Figure 3). This is possibly because this is a new virus, diagnosed seven years ago, and there has not been much research published on it [50]. The remainder of the viruses in Figure 3 have either been diagnosed in a combination of free-ranging and captive animals, e.g., *Foot and mouth disease virus*, *African swine fever virus*, or only in free-ranging ungulates, e.g., *Akabane orthobunyavirus*, *Bluetongue virus*. It appeared that viruses that were diagnosed in both free-ranging and captive animals, e.g., *Foot and mouth disease virus*, *African swine fever virus*, were the viruses that seemed to have the most publications reporting on them, likely because these viruses were the ones of major interest in the wildlife and livestock agricultural sectors. It is recommended that future research in this field be focused on *African elephant polyomavirus 1*. However, currently, the virus does not seem to bear severe consequences or risks for the health of free-ranging elephants; therefore, passive or low-grade active surveillance can be performed in addition to other diseases being researched/surveyed to maximise resource use. An additional recommendation is to perform research dedicated to investigating *Akabane orthobunyavirus* and its relationship with black and white rhinoceros as it may be of interest to the conservation of these endangered species.

#### 4.1.3. Specific Ungulates Affected by Viruses

Viruses or antibodies were found in a wide variety of African ungulates (Table 4). Seventeen percent of diagnosed viruses/viral diseases in African ungulates were represented by the African buffalo, which was by far the ungulate diagnosed with the most viruses. African buffalo are susceptible to 16 of the 32 viruses mentioned in this study. This may be because African buffalo are widely spread across sub-Saharan Africa; they are one of the most studied wild African ungulates due to their association with foot and mouth disease and possibly because they are reasonably easy to locate, immobilise and sample.

Blue wildebeest represented 7% of diagnosed viruses/viral diseases in African ungulates, with a high number of publications reporting on *Alcelaphine gammaherpesvirus 1*. This indicates the relationship between the blue wildebeest and *Alcelaphine gammaherpesvirus 1*, with blue wildebeest being the reservoir host for this virus [37]. Blue wildebeest are also an ungulate species very commonly found throughout sub-Saharan Africa.

Impala represented 6% of diagnosed viruses/viral diseases in African ungulates. Once again, this may be because foot and mouth disease was the viral disease which had the most publications reporting on it. Impala are also widely spread across sub-Saharan Africa.

Warthogs also represented 6% of diagnosed viruses/viral diseases in African ungulates. This may be because the warthog is the wild reservoir host for *African swine fever virus* and African swine fever was the viral disease with the second highest number of publications reported [30]. Warthogs also inhabit vast areas of sub-Saharan Africa.

#### 4.1.4. Geographical Distribution of Viruses

All the publications in this study reported on viruses/viral diseases in ungulates from sub-Saharan Africa. The geographical distribution map indicates that the majority of the publications reported on viruses/viral diseases in ungulates in Southern and Eastern Africa, with a small proportion from Western Africa and none from Central or Northern Africa. Several factors may contribute to this distribution. The most likely factor is the concentration of research institutions and funding available in each of these geographical regions of Africa, with higher concentrations present in more developed African countries. An additional factor is that wildlife has been a major tourist attraction in Southern and Eastern African countries for many years. This has resulted in wildlife being seen as a valuable resource and so keeping wildlife healthy was of economic benefit. Another factor could be past or ongoing war and conflict. Countries severely affected by war have lower numbers of publications because wildlife numbers are often decimated during war and scientists are less likely to work in countries where their lives are in danger [10,51]. Examples of countries affected by war include Angola and Mozambique. There were zero publications reporting on viruses/viral diseases in ungulates from Angola and only one from Mozambique, despite both countries being in Southern Africa. Furthermore, several studies originated from continents besides Africa, namely Europe and North America. These studies were included for thoroughness and pertain to viral diseases in wild African ungulates in captivity, but their data were not used to calculate percentages of publications dealing with free-ranging compared to captive ungulates.

#### 4.1.5. Viruses which Seem to Be “Under-Studied”

Several of the 32 viruses reported to infect African ungulates are classified as high-impact viruses because they have a significant negative impact on the health and lives of animals and humans [10,25] and are listed as notifiable diseases to the OIE [26]. The high-impact viral diseases diagnosed in African ungulates that are of significance in the African context are as follows:

- Foot and mouth disease
- African swine fever
- Rift Valley fever
- Bluetongue
- Rabies
- Lumpy skin disease
- Peste des petits ruminants
- African horse sickness

Interestingly, a virus species that affects a wide range of African ungulates does not necessarily classify that particular virus as high-impact. For example, of the top five virus species affecting the widest ranges of African ungulates, *Foot and mouth disease virus*, *Pestivirus A/B* and *Bovine alphaherpesvirus 1* represent some of the high-impact viruses and only *Foot and mouth disease virus* is of significance in the African context.

Certain diseases, such as peste des petits ruminants, which can have a significant impact on wildlife, do not seem to receive as much attention as they should. It would be helpful to have a “Wildlife disease and infection” category, similar to the other categories on the list, added to OIE-listed diseases. For example, Rift Valley fever and infection with *Elephant polyomavirus 1* could be listed under the new category. This is an important consideration, especially to allow future conservation efforts and campaigns to take diseases into account, as infectious diseases are becoming more prevalent in wildlife populations with the intensification of agriculture and the increased amount of wildlife/livestock/human interactions [3]. The OIE-listed diseases pertain to the World Trade Organisation; if certain diseases are threatening the conservation and/or associated economy of a wildlife species, then, ideally, trade that may spread that disease should be halted.

A clear knowledge gap is highlighted in research focusing on *Lumpy skin disease virus*, *Small ruminant morbillivirus* and *African horse sickness virus*. The reason for the under-reporting of research on these three diseases may be due to the difficulty of testing and surveillance for disease in free-ranging African ungulates. For example, game rangers may come across a dead animal and if the carcass is fresh, samples may be collected. However, synthesising a case report from limited information is particularly challenging and unlikely to be published in a peer-reviewed scientific journal unless it is of great significance. Another reason for which these diseases may be under-reported is that lumpy skin disease and African horse sickness do not cause significant clinical disease in African ungulates. In addition, disease research focuses mainly on livestock, instead of wildlife, because agriculture and food production play a major role in the economies of countries across the globe. Therefore, many publications discussing diseases in wildlife is due to the disease being important in livestock. A good example of this is foot and mouth disease, which is a very important disease in livestock but much less so in wildlife. Nevertheless, these diseases are of great significance in the context of livestock health and, given that African ungulates may play a role in the epidemiology of these diseases, it is important that these diseases are strongly considered as research topics in the future. 

### 4.2. Limitations and Constraints

This research study has several limitations. In the first instance, it was predisposed to database bias as only three multidisciplinary databases were interrogated during the search process and the search strategy delivered mainly veterinary-related articles. If other databases were interrogated, additional publications may have been obtained [52]. In addition, making use of publications from databases alone also predisposes the research study to temporal bias that may have resulted in the exclusion of older publication not available in the databases. Given that this scoping review was based on scientific publications, it was predisposed to publication bias affected by author’s career status, institution, language, country, study outcome, research topic, research sponsor and timeline [53,54]. This study was also prone to spatial bias as research and publication concentrate in more developed countries, e.g., Zimbabwe (prior to 1985), South Africa, Namibia, Botswana, Kenya and Tanzania, due to being correlated to economic indices [51]. Geographical bias also plays a role, as there was an underrepresentation of publications from specific regions in Africa, particularly Northern, Western and Central Africa, and may suggest limited resources and capacity for wildlife surveillance in these areas.

Constraints were necessary and were put into place to maintain a practicable scope for this research study. The importance of viral diseases in terms of economic, health and conservation impacts was not quantified. Only viral diseases diagnosed in African ungulates were relevant and deemed sufficiently extensive to satisfy the objectives of the scoping review. All indigenous African ungulates were listed and included in the search. This may have resulted in the exclusion of a very rare ungulate species that may not have been identified yet but this scenario is highly unlikely.

Camels were excluded from the list of indigenous African ungulates because the majority of camels present in Africa are domesticated or feral and were introduced to Africa. Therefore, camels are not African ungulates by definition.

Categorising wildlife into captive, semi-captive and free-ranging could not be achieved during the search process, given the constraints of the methodology. Hence, only two categories, namely captive and free-ranging wildlife, were set.

Given the limitations of this research study, it is necessary to highlight that the findings presented in this discussion indicate the perceived emphasis placed on different viruses and viral diseases by scientists and should not be perceived as the incidence or occurrence of viral diseases in African ungulates. Furthermore, results were presented in a quantitative format. In some instances, this may imply that a virus with the highest number of detections in several host species is the most important one. However, in reality, this may not be the case. For example, a rarely detected virus may be considered significant as a future research focus as it may pose a significant threat to the conservation of an ungulate species. Existing knowledge of the ecosystem dynamics for many multi-host viral diseases is deficient [55]; therefore, it is recommended that research is performed in this field, including quantitative research focusing on viral diseases in African ungulates, to further clarify the role of wildlife in the epidemiology of these diseases, and, moreover, to provide evidence of the importance of these diseases at the wildlife/livestock interface.

## 5. Conclusions

The viral diseases of African ungulates that have received the most attention over the past six decades have been highlighted, as well as the diseases that have not received adequate attention. There are a variety of viruses which have been diagnosed in African ungulates and the large majority of African ungulates included in the study have had one or more viruses or viral diseases associated with them.

It is anticipated that these findings will be valuable to policymakers, funding bodies, researchers and other stakeholders who need an understanding of viral diseases in African ungulates. Research opportunities in this field will allow them to make informed decisions about investment in future research projects and animal health policies and protocols. It is recommended that governments and research institutions offer more funding to investigate and report viral diseases of greater clinical and zoonotic significance, such as rabies and Rift Valley fever. This is especially important in the current climate of emerging diseases and the related overflow of disease from wild to domestic animals and from animals, both wild and domestic, to humans. A further recommendation is for appropriate One Health approaches to be adopted for investigating, controlling, managing and preventing diseases [3]. This is especially true for diseases such as African swine fever and Rift Valley fever, where human actions, poor biosecurity and natural weather changes play a major role in the transmission of diseases [3,30,33]. Diseases which may threaten the conservation of certain wildlife species also require focused attention. In order to keep track of these diseases, it would be helpful to add a “Wildlife disease and infection” category to OIE-listed diseases, the reason being that if certain diseases are threatening the conservation and/or associated economy of a wildlife species, then ideally, trade that may spread that disease should be halted.

Viral diseases, as a whole, are of great significance and require extra attention in the future as they make up a large proportion of emerging infectious diseases and can often infect multiple hosts [1,28]. Hence, the viruses and viral diseases diagnosed in African ungulates are of significance, particularly at the wildlife/livestock interface, and many of them have the potential to become emerging wildlife diseases.

## Figures and Tables

**Table 1:** The proportion of different types of publications reporting on viruses/viral diseases in African ungulates.

Publication Type	Count of Publications	Percentage of Total Number of Publications (145)
Assay (molecular) development	1	1
Assay (antibody) development	4	3
Experiment	25	17
Phylogenetic study	29	20
Case/outbreak report	31	21
Surveillance	58	40
Total	148	

**Table 2:** Number of publications reporting on each virus in African ungulates.

Virus	Publication Count
*Foot and mouth disease virus*	46
*African swine fever virus*	20
*Alcelaphine gammaherpesvirus 1*	17
*Rift Valley fever phlebovirus*	9
*Elephantid betaherpesvirus 1/4/5*	8
*Pestivirus A/B*	7
*Bluetongue virus*	5
*Bovine alphaherpesvirus 2*	5
*Rabies lyssavirus*	5
*Rinderpest morbillivirus*	5
*African horse sickness virus*	4
*Bovine alphaherpesvirus 1*	4
*Cardiovirus A*	4
*Equid alphaherpesvirus 1*	4
*Akabane orthobunyavirus*	3
*Bovine respirovirus 3*	3
*Lumpy skin disease virus*	3
*Bovine fever ephemerovirus*	2
*Bovine gammaherpesvirus 4*	2
*Deltapapillomavirus 4*	2
*Epizootic hemorrhagic disease virus*	2
*Equid alphaherpesvirus 9*	2
*Ovine gammaherpesvirus 2*	2
*African elephant polyomavirus 1*	1
*Alphaarterivirus equid*	1
*Bovine mastadenovirus*	1
*Equid alphaherpesvirus 4*	1
*Equine encephalosis virus*	1
*Hippotragine gammaherpesvirus 1*	1
*Pestivirus C*	1
*Small ruminant morbillivirus*	1
*Wesselsbron virus*	1
Total	173

**Table 3:** Number of publications indicating detection of viral antigen/antibody in each species of African ungulate.

	Total	*African horse sickness virus*	*Bluetongue virus*	*Epizootic hemorrhagic disease virus*	*Foot-and-mouth disease virus*	*Cardiovirus A*	*Small ruminant morbillivirus*	*Bovine respirovirus 3*	*Equid alphaherpesvirus 1*	*Equid alphaherpesvirus 9*	*Rinderpest morbillivirus*	*African swine fever virus*	*Bovine mastadenovirus*	*Alcelaphine gammaherpesvirus 1*	*Ovine gammaherpesvirus 2*	*Equine encephalosis virus*	*Rift Valley fever phlebovirus*	*Bovine alphaherpesvirus 1*	*Alphaarterivirus equid*	*Lumpy skin disease virus*	*Wesselsbron virus*	*Akabane orthobunyavirus*	*Bovine fever ephemerovirus*	*Pestivirus A/B*	*Equid alphaherpesvirus 4*	*Elephantid betaherpesvirus 1/4/5*	*Bovine gammaherpesvirus 4*	*Pestivirus C*	*Rabies lyssavirus*	*Hippotragine gammaherpesvirus 1*	*African elephant polyomavirus 1*	*Deltapapillomavirus 4*	*Bovine alphaherpesvirus 2*
**Total**	466	7	28	3	94	6	1	26	11	3	11	24	1	37	2	3	20	28	1	7	15	14	14	32	1	8	2	2	10	1	1	4	49
**Ass, African wild**	1								1																								
**Bontebok/Blesbok**	7		1											1				1			1		1	1									1
**Buffalo, African/Cape**	79		3		41			2			4		1	1	1		8	3		2	1	2	1	2			2						5
**Bushbuck**	8				1						1							1			1		1										3
**Bushpig**	9				1			1				6																1					
**Dik-dik, Kirk’s**	1																							1									
**Duiker**	1				1																												
**Duiker, Blue**	1				1																												
**Duiker, Common**	3						1																	1					1				
**Eland, Common**	20		1		2			2			1						1	1		1	1		1	3					4				2
**Eland, Giant**	1				1																												
**Elephant, African**	20	2	1		1	3										1	1				1					8					1		1
**Gazelle, Grant’s**	3				2																												1
**Gazelle, Thomson’s**	3				2												1																
**Gemsbok**	13		2					2						3				1			1			2									2
**Gerenuk**	1																1																
**Giant forest hog**	1																																1
**Giraffe**	12		1		1	1					1						1				1			2								1	3
**Hartebeest**	18		3		3	1		1						2			1	1			1	1	1	1									2
**Hippopotamus**	4													1				1															2
**Hog, Red river**	3				1							2																					
**Ibex, Nubian**	1													1																			
**Impala**	29		3		14	1		1			1			1			1	1		1			1	1									3
**Kob**	3				1			1										1															
**Kudu, Greater**	22				5			1						2				2			1		1	3					5				2
**Kudu, Lesser**	3										2						1																
**Lechwe**	5		1					1						1				1						1									
**Nyala**	1																							1									
**Oribi**	2				1			1																									
**Oryx, East African**	2													1																			1
**Oryx, Scimitar-horned**	1													1																			
**Reedbuck, Bohor**	1				1																												
**Reedbuck, Southern**	5							1						1				1						1									1
**Rhebok, Grey**	1														1																		
**Rhinoceros, Black**	14	1	1	1				1	2	1							1	1			1	2		1									1
**Rhinoceros, White**	16	1	2	2				1	2								1	2			1	2		1									1
**Roan antelope**	5				1			1										1												1			1
**Sable antelope**	8		1					1						2				1						1								1	1
**Sheep, Barbary**	1																							1									
**Springbok**	11		1					2												1	1	1	1	2									2
**Tsessebe, Bangweulu**	2		1											1																			
**Tsessebe, Common/Topi**	15		2		4			1						2				2				1	1										2
**Warthog, Common**	26				4			1				15					1					1		1				1					2
**Warthog, Desert**	1											1																					
**Waterbuck**	16		1		2			1						1				2				1	2	2									4
**Wildebeest, Black**	12		1					1						3				1		1	1	1	1	1									1
**Wildebeest, Blue**	34		2		3			2			1			12				3		1	1	1	2	2									4
**Zebra, Grévy’s**	1								1																								
**Zebra, Mountain**	5	1							2							1																1	
**Zebra, Plains**	14	2							3	2						1	1		1		1	1			1							1	

**Table 4:** List of African ungulate species, in alphabetical order, reported to have virus antigen and/or antibodies detected.

Ungulate—Common Name	Ungulate—Genus and Species
Ass, African wild	*Equus africanus*
Bontebok/Blesbok	*Damaliscus pygargus*
Buffalo, African/Cape	*Syncerus caffer*
Bushbuck	*Tragelaphus sylvaticus*
Bushpig	*Potamochoerus larvatus*
Dik-dik, Kirk’s	*Madoqua kirkii*
Duiker	*Cephalophus silvicultor*
Duiker, Blue	*Philantomba monticola*
Duiker, Common	*Sylvicapra grimmia*
Eland, Common	*Taurotragus oryx*
Eland, Giant	*Taurotragus derbianus*
Elephant, African	*Loxodonta africana*
Gazelle, Grant’s	*Gazella granti*
Gazelle, Thomson’s	*Eudorcas thomsonii*
Gemsbok	*Oryx gazelle*
Gerenuk	*Litocranius walleri*
Giant forest hog	*Hylochoerus meinertzhageni*
Giraffe	*Giraffa camelopardalis*
Hartebeest	*Alcelaphus buselaphus*
Hippopotamus	*Hippopotamus amphibius*
Hog, Red river	*Potamochoeus porcus*
Ibex, Nubian	*Capra nubiana*
Impala	*Aepyceros melampus*
Kob	*Kobus kob*
Kudu, Greater	*Tragelaphus strepsiceros*
Kudu, Lesser	*Tragelaphus imberbis*
Lechwe	*Kobus leche*
Nyala	*Tragelaphus angasii*
Oribi	*Ourebia ourebi*
Oryx, East African	*Oryx beisa*
Oryx, Scimitar-horned	*Oryx dammah*
Reedbuck, Bohor	*Redunca*
Reedbuck, Southern	*Redunca arundinum*
Rhebok, Grey	*Pelea capreolus*
Rhinoceros, Black	*Diceros bicornis*
Rhinoceros, White	*Ceratotherium simun*
Roan antelope	*Hippotragus equinus*
Sable antelope	*Hippotragus niger*
Sheep, Barbary	*Ammotragus lervia*
Springbok	*Antidorcas masupialis*
Tsessebe, Bangweulu	*Damaliscus superstes*
Tsessebe, Common/Topi	*Damaliscus lunatus*
Warthog, Common	*Phacochoerus africanus*
Warthog, Desert	*Phacochoerus aethiopicus*
Waterbuck	*Kobus ellipsiprymnus*
Wildebeest, Black	*Connochaetes gnou*
Wildebeest, Blue	*Connochaetes taurinus*
Zebra, Grévy’s	*Equus grevyi*
Zebra, Mountain	*Equus zebra*
Zebra, Plains	*Equus quagga*

**Table 5:** (**a**) Viruses diagnosed in ungulates per African country. (**b**) Country codes indicated are as described in the ISO 3166 international standard.

(**a**)																															
	***African horse sickness virus***	***African swine fever virus***	***Akabane orthobunyavirus***	***Alcelaphine gammaherpesvirus 1***	***Alphaarterivirus equid***	***Bluetongue virus***	***Bovine alphaherpesvirus 1***	***Bovine alphaherpesvirus 2***	***Bovine fever ephemerovirus***	***Bovine gammaherpesvirus 4***	***Bovine mastadenovirus***	***Bovine respirovirus 3***	***Cardiovirus A***	***Deltapapillomavirus 4***	***Elephantid betaherpesvirus 1/4/5***	***Epizootic hemorrhagic disease virus***	***Equid alphaherpesvirus 1***	***Equid alphaherpesvirus 4***	***Equid alphaherpesvirus 9***	***Equine encephalosis virus***	***Foot-and-mouth disease virus***	***Lumpy skin disease virus***	***Ovine gammaherpesvirus 2***	***Pestivirus A/B***	***Pestivirus C***	***Rabies lyssavirus***	***Rift Valley fever phlebovirus***	***Rinderpest morbillivirus***	***Small ruminant morbillivirus***	***Wesselsbron virus***	**Total**
**BEN**	0	0	0	0	0	0	0	0	0	0	0	0	0	0	0	0	0	0	0	0	1	0	0	0	0	0	0	0	0	0	**1 **
**BFA**	0	0	0	0	0	0	0	0	0	0	0	0	0	0	0	0	0	0	0	0	1	0	0	0	0	0	0	0	0	0	**1 **
**BWA**	0	1	0	0	0	1	1	1	0	0	0	1	0	0	0	0	0	0	0	0	7	0	0	0	0	0	0	0	0	0	**12 **
**CAF**	0	0	0	0	0	0	0	0	0	0	0	0	0	0	0	0	0	0	0	0	1	0	0	0	0	0	0	0	0	0	**1 **
**COD**	0	0	0	0	0	0	0	0	0	0	0	0	0	0	0	0	0	0	0	0	2	0	0	0	0	0	0	0	0	0	**2 **
**GAB**	0	0	0	0	0	0	0	0	0	0	0	0	0	0	0	0	0	0	0	0	1	0	0	0	0	0	0	0	0	0	**1 **
**KEN**	1	6	1	3	0	1	1	2	0	2	0	1	0	0	1	1	0	0	0	0	3	1	0	1	0	0	3	4	0	0	**32 **
**MOZ**	0	0	0	0	0	0	0	0	0	0	0	0	0	0	0	0	0	0	0	0	1	0	0	0	0	0	0	0	0	0	**1 **
**MWI**	0	1	0	0	0	0	0	0	0	0	0	0	0	0	0	0	0	0	0	0	1	0	0	0	0	0	0	0	0	0	**2 **
**NAM**	1	3	1	0	0	1	0	1	0	0	0	2	0	0	0	1	2	0	1	0	5	0	0	2	0	5	0	0	0	0	**25 **
**NGA**	0	3	0	0	0	0	0	0	0	0	0	0	0	0	0	0	0	0	0	0	1	0	0	0	0	0	0	0	1	0	**5 **
**SWZ**	0	1	0	0	0	0	0	0	0	0	0	0	0	0	0	0	0	0	0	0	0	0	0	0	0	0	0	0	0	0	**1 **
**TCD**	0	0	0	0	0	0	0	1	0	0	0	1	0	0	0	0	0	0	0	0	1	0	0	0	0	0	0	0	0	0	**3 **
**TZA**	0	4	1	3	1	1	1	3	1	1	0	0	0	0	0	0	2	1	2	0	5	0	0	2	0	0	0	3	0	0	**31 **
**UGA**	0	3	0	0	0	0	0	2	0	1	0	1	0	0	0	0	0	0	0	0	7	0	0	0	0	0	1	0	0	0	**15 **
**ZAF**	3	6	2	7	0	3	2	2	1	1	0	3	2	2	2	2	2	0	0	1	22	2	1	2	1	1	7	0	0	1	**78 **
**ZMB**	0	0	0	0	0	0	0	1	0	0	0	1	0	0	0	0	0	0	0	0	7	0	0	0	0	0	0	0	0	0	**9 **
**ZWE**	0	3	0	0	0	0	0	1	0	0	1	1	0	0	0	0	0	0	0	0	14	0	0	0	0	0	0	0	0	0	**20 **
**Total**	**5 **	**31 **	**5 **	**13 **	**1 **	**7 **	**5 **	**14 **	**2 **	**5 **	**1 **	**11 **	**2 **	**2 **	**3 **	**4 **	**6 **	**1 **	**3 **	**1 **	**80 **	**3 **	**1 **	**7 **	**1 **	**6 **	**11 **	**7 **	**1 **	**1 **	**240 **
(**b**)																															
Country Abbreviation																Country															
BEN																Benin															
BWA																Botswana															
BFA																Burkina Faso															
CAF																Central African Republic															
TCD																Chad															
COD																Democratic Republic of the Congo															
SWZ																Eswatini															
GAB																Gabon															
KEN																Kenya															
MWI																Malawi															
MOZ																Mozambique															
NAM																Namibia															
NGA																Nigeria															
ZAF																South Africa															
TZA																Tanzania															
UGA																Uganda															
ZMB																Zambia															
ZWE																Zimbabwe															

**Table 6:** The number of African ungulate species affected by a virus family/genus/species.

Family	Count	Genus	Count	Species	Count
*Adenoviridae*	1	*Mastadenovirus*	1	*Bovine mastadenovirus*	1
*Arteriviridae*	1	*Alphaarterivirus*	1	*Alphaarterivirus equid*	1
*Asfarviridae*	4	*Asfivirus*	4	*African swine fever virus*	4
*Flaviviridae*	26	*Flavivirus*	15	*Wesselsbron virus*	15
		*Pestivirus*	23	*Pestivirus A/B*	22
				*Pestivirus C*	2
*Herpesviridae*	35	*Macavirus*	20	*Alcelaphine gammaherpesvirus 1*	18
				*Ovine gammaherpesvirus 2*	2
				*Hippotragine gammaherpesvirus 1*	1
		*Proboscivirus*	1	*Elephantid betaherpesvirus 1/4/5*	1
		*Rhadinovirus*	1	*Bovine gammaherpesvirus 4*	1
		*Simplexvirus*	24	*Bovine alphaherpesvirus 2*	24
		*Varicellovirus*	24	*Equid alphaherpesvirus 1*	6
				*Equid alphaherpesvirus 9*	2
				*Bovine alphaherpesvirus 1*	20
				*Equid alphaherpesvirus 4*	1
*Papillomaviridae*	4	*Deltapapillomavirus*	4	*Deltapapillomavirus 4*	4
*Paramyxoviridae*	25	*Morbillivirus*	7	*Small ruminant morbillivirus*	1
				*Rinderpest morbillivirus*	6
		*Respirovirus*	21	*Bovine respirovirus 3*	21
*Peribunyaviridae*	11	*Orthobunyavirus*	11	*Akabane orthobunyavirus*	11
*Phenuiviridae*	13	*Phlebovirus*	13	*Rift Valley fever phlebovirus*	13
*Picornaviridae*	23	*Aphthovirus*	23	*Foot and mouth disease virus*	23
		*Cardiovirus*	4	*Cardiovirus A*	4
*Polyomaviridae*	1	*Polyomavirus*	1	*African elephant polyomavirus 1*	1
*Poxviridae*	6	*Capripoxvirus*	6	*Lumpy skin disease virus*	6
*Reoviridae*	20	*Orbivirus*	20	*African horse sickness virus*	5
				*Bluetongue virus*	18
				*Epizootic hemorrhagic disease virus*	2
				*Equine encephalosis virus*	3
*Rhabdoviridae*	13	*Ephemerovirus*	12	*Bovine fever ephemerovirus*	12
		*Lyssavirus*	3	*Rabies lyssavirus*	3

**Figure A1:**
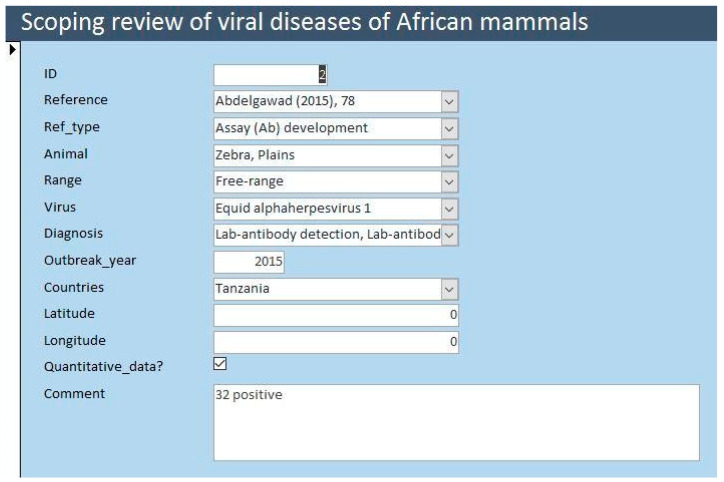
Screenshot of a Microsoft Access database form used to extract data from publications.

## Data Availability

Data sharing not applicable. No new data were created or analyzed in this study. Data sharing is not applicable to this article.

## Appendix A

**Table A1:** Preferred Reporting Items for Systematic Reviews and Meta-Analyses extension for scoping reviews (PRISMA-ScR) checklist indicating the page on which the specific item is reported.

Section	ITEM	PRISMA-ScR Checklist Item	Reported on Page #
**Title**			
Title	1	Identify the report as a scoping review.	1
**Abstract**			
Structured summary	2	Provide a structured summary that includes (as applicable): background, objectives, eligibility criteria, sources of evidence, charting methods, results, and conclusions that relate to the review questions and objectives.	1
**Introduction**			
Rationale	3	Describe the rationale for the review in the context of what is already known. Explain why the review questions/objectives lend themselves to a scoping review approach.	1
Objectives	4	Provide an explicit statement of the questions and objectives being addressed with reference to their key elements (e.g., population or participants, concepts, and context) or other relevant key elements used to conceptualize the review questions and/or objectives.	2
**Methods**			
Protocol and registration	5	Indicate whether a review protocol exists; state if and where it can be accessed (e.g., a Web address); and if available, provide registration information, including the registration number.	3
Eligibility criteria	6	Specify characteristics of the sources of evidence used as eligibility criteria (e.g., years considered, language, and publication status), and provide a rationale.	3
Information sources *	7	Describe all information sources in the search (e.g., databases with dates of coverage and contact with authors to identify additional sources), as well as the date the most recent search was executed.	4
Search	8	Present the full electronic search strategy for at least 1 database, including any limits used, such that it could be repeated.	4
Selection of sources of evidence †	9	State the process for selecting sources of evidence (i.e., screening and eligibility) included in the scoping review.	5
Data charting process ‡	10	Describe the methods of charting data from the included sources of evidence (e.g., calibrated forms or forms that have been tested by the team before their use, and whether data charting was done independently or in duplicate) and any processes for obtaining and confirming data from investigators.	5
Data items	11	List and define all variables for which data were sought and any assumptions and simplifications made.	6
Critical appraisal of individual sources of evidence §	12	If done, provide a rationale for conducting a critical appraisal of included sources of evidence; describe the methods used and how this information was used in any data synthesis (if appropriate).	7
Synthesis of results	13	Describe the methods of handling and summarizing the data that were charted.	7
**Results**			
Selection of sources of evidence	14	Give numbers of sources of evidence screened, assessed for eligibility, and included in the review, with reasons for exclusions at each stage, ideally using a flow diagram.	7
Characteristics of sources of evidence	15	For each source of evidence, present characteristics for which data were charted and provide the citations.	8
Critical appraisal within sources of evidence	16	If done, present data on critical appraisal of included sources of evidence (see item 12).	N/A
Results of individual sources of evidence	17	For each included source of evidence, present the relevant data that were charted that relate to the review questions and objectives.	N/A
Synthesis of results	18	Summarize and/or present the charting results as they relate to the review questions and objectives.	8
**Discussion**			
Summary of evidence	19	Summarize the main results (including an overview of concepts, themes, and types of evidence available), link to the review questions and objectives, and consider the relevance to key groups.	17
Limitations	20	Discuss the limitations of the scoping review process.	21
Conclusions	21	Provide a general interpretation of the results with respect to the review questions and objectives, as well as potential implications and/or next steps.	22
**Funding**			
Funding	22	Describe sources of funding for the included sources of evidence, as well as sources of funding for the scoping review. Describe the role of the funders of the scoping review.	49

JBI = Joanna Briggs Institute; PRISMA-ScR = Preferred Reporting Items for Systematic reviews and Meta-Analyses extension for Scoping Reviews. * Where sources of evidence (see second footnote) are compiled from, such as bibliographic databases, social media platforms and websites. † A more inclusive/heterogeneous term used to account for the different types of evidence or data sources (e.g., quantitative and/or qualitative research, expert opinion and policy documents) that may be eligible in a scoping review as opposed to only studies. This is not to be confused with *information sources* (see first footnote). ‡ The frameworks by Arksey and O’Malley (6) and Levac and colleagues (7) and the JBI guidance (4, 5) refer to the process of data extraction in a scoping review as data charting. § The process of systematically examining research evidence to assess its validity, results and relevance before using it to inform a decision. This term is used for items 12 and 19 instead of “risk of bias” (which is more applicable to systematic reviews of interventions) to include and acknowledge the various sources of evidence that may be used in a scoping review (e.g., quantitative and/or qualitative research, expert opinion and policy document). From: Tricco, A.C.; Lillie, E.; Zarin, W.; O’Brien, K.K.; Colquhoun, H.; Levac, D. et al. PRISMA Extension for Scoping Reviews (PRISMA-ScR): Checklist and Explanation. *Ann. Intern. Med.* 2018, *169*, 467–473, doi: 10.7326/M18-0850.

## Appendix B

### Appendix B.1. Adapted for Scopus

(TITLE-ABS-KEY (africa*) AND TITLE-ABS-KEY (virus OR viral)) AND (TITLE-ABS-KEY (loxodonta OR “african elephant” OR giraff* OR syncerus OR “african buffalo” OR “cape buffalo” OR hippopotamus OR choeropsis OR rhinoceros OR ceratotherium OR diceros OR “equus zebra” OR “equus africanus” OR grevyi OR quagga OR phacochoerus OR warthog OR potamochoerus OR bushpig OR “red river hog” OR aepyceros OR impala OR alcelaphus OR hartebees* OR connochaetes OR wildebees* OR damaliscus OR tsessebe OR bonteb* OR blesb* OR antidorcas OR springb* OR raphicerus OR steenb* OR grysbok OR tragelaphus OR kudu OR koedoe OR nyala OR bongo OR bushbuck OR bosbok OR sitatunga OR taurotragus OR eland OR hippotragus OR sable OR roan OR oryx OR gemsb* OR pelea OR rheb* OR redunca OR reedbuck OR rietbok OR “kobus ellipsiprymnus” OR waterb* OR “kobus leche” OR lechwe OR “kobus kob” OR “kobus vardonii” OR puku OR cephalophus OR sylvicapra OR philantomba OR duiker OR oreotragus OR klipspringer OR spekei OR leptoceros OR “Gazella dorcas” OR eudorcas OR nanger OR addax OR “capra nubiana” OR “nubian ibex” OR beatragus OR hirola OR ammotragus OR “barbary sheep” OR dorcatragus OR madoqua OR “dik-dik” OR okapia OR okapi OR neotragus OR “royal antelope” OR suni OR litocranius OR gerenuk OR hyemoschus OR chevrotain OR ourebia OR oribi)) AND NOT (TITLE-ABS-KEY (beetle OR arthropod OR oryctes OR nudivirus OR javan OR sumatran OR “one horned” OR snake OR chicken* OR human* OR Newcastle OR arabi* OR tragus OR aquaculture OR waterborne)).

### Appendix B.2. Adapted for Wildlife and Ecology Studies Worldwide

TI (Africa*) AND TI (virus OR viral) AND TI (loxodonta OR “african elephant” OR giraff* OR syncerus OR “african buffalo” OR “cape buffalo” OR hippopotamus OR choeropsis OR rhinoceros OR ceratotherium OR diceros OR “equus zebra” OR “equus africanus” OR grevyi OR quagga OR phacochoerus OR warthog OR potamochoerus OR bushpig OR “red river hog” OR aepyceros OR impala OR alcelaphus OR hartebees* OR connochaetes OR wildebees* OR damaliscus OR tsessebe OR bonteb* OR blesb* OR antidorcas OR springb* OR raphicerus OR steenb* OR grysbok OR tragelaphus OR kudu OR koedoe OR nyala OR bongo OR bushbuck OR bosbok OR sitatunga OR taurotragus OR eland OR hippotragus OR sable OR roan OR oryx OR gemsb* OR pelea OR rheb* OR redunca OR reedbuck OR rietbok OR “kobus ellipsiprymnus” OR waterb* OR “kobus leche” OR lechwe OR “kobus kob” OR “kobus vardonii” OR puku OR cephalophus OR sylvicapra OR philantomba OR duiker OR oreotragus OR klipspringer OR spekei OR leptoceros OR “Gazella dorcas” OR eudorcas OR nanger OR addax OR “capra nubiana” OR “nubian ibex” OR beatragus OR hirola OR ammotragus OR “barbary sheep” OR dorcatragus OR madoqua OR “dik-dik” OR okapia OR okapi OR neotragus OR “royal antelope” OR suni OR litocranius OR gerenuk OR hyemoschus OR chevrotain OR ourebia OR oribi) NOT TI (beetle OR arthropod OR oryctes OR nudivirus OR javan OR sumatran OR “one horned” OR snake OR chicken* OR human* OR Newcastle OR arabi* OR tragus OR aquaculture OR waterborne) OR AB (Africa*) AND AB (virus OR viral) AND AB (loxodonta OR “african elephant” OR giraff* OR syncerus OR “african buffalo” OR “cape buffalo” OR hippopotamus OR choeropsis OR rhinoceros OR ceratotherium OR diceros OR “equus zebra” OR “equus africanus” OR grevyi OR quagga OR phacochoerus OR warthog OR potamochoerus OR bushpig OR “red river hog” OR aepyceros OR impala OR alcelaphus OR hartebees* OR connochaetes OR wildebees* OR damaliscus OR tsessebe OR bonteb* OR blesb* OR antidorcas OR springb* OR raphicerus OR steenb* OR grysbok OR tragelaphus OR kudu OR koedoe OR nyala OR bongo OR bushbuck OR bosbok OR sitatunga OR taurotragus OR eland OR hippotragus OR sable OR roan OR oryx OR gemsb* OR pelea OR rheb* OR redunca OR reedbuck OR rietbok OR “kobus ellipsiprymnus” OR waterb* OR “kobus leche” OR lechwe OR “kobus kob” OR “kobus vardonii” OR puku OR cephalophus OR sylvicapra OR philantomba OR duiker OR oreotragus OR klipspringer OR spekei OR leptoceros OR “Gazella dorcas” OR eudorcas OR nanger OR addax OR “capra nubiana” OR “nubian ibex” OR beatragus OR hirola OR ammotragus OR “barbary sheep” OR dorcatragus OR madoqua OR “dik-dik” OR okapia OR okapi OR neotragus OR “royal antelope” OR suni OR litocranius OR gerenuk OR hyemoschus OR chevrotain OR ourebia OR oribi) NOT AB (beetle OR arthropod OR oryctes OR nudivirus OR javan OR sumatran OR “one horned” OR snake OR chicken* OR human* OR Newcastle OR arabi* OR tragus OR aquaculture OR waterborne) OR KA (Africa*) AND KA (virus OR viral) AND KA (loxodonta OR “african elephant” OR giraff* OR syncerus OR “african buffalo” OR “cape buffalo” OR hippopotamus OR choeropsis OR rhinoceros OR ceratotherium OR diceros OR “equus zebra” OR “equus africanus” OR grevyi OR quagga OR phacochoerus OR warthog OR potamochoerus OR bushpig OR “red river hog” OR aepyceros OR impala OR alcelaphus OR hartebees* OR connochaetes OR wildebees* OR damaliscus OR tsessebe OR bonteb* OR blesb* OR antidorcas OR springb* OR raphicerus OR steenb* OR grysbok OR tragelaphus OR kudu OR koedoe OR nyala OR bongo OR bushbuck OR bosbok OR sitatunga OR taurotragus OR eland OR hippotragus OR sable OR roan OR oryx OR gemsb* OR pelea OR rheb* OR redunca OR reedbuck OR rietbok OR “kobus ellipsiprymnus” OR waterb* OR “kobus leche” OR lechwe OR “kobus kob” OR “kobus vardonii” OR puku OR cephalophus OR sylvicapra OR philantomba OR duiker OR oreotragus OR klipspringer OR spekei OR leptoceros OR “Gazella dorcas” OR eudorcas OR nanger OR addax OR “capra nubiana” OR “nubian ibex” OR beatragus OR hirola OR ammotragus OR “barbary sheep” OR dorcatragus OR madoqua OR “dik-dik” OR okapia OR okapi OR neotragus OR “royal antelope” OR suni OR litocranius OR gerenuk OR hyemoschus OR chevrotain OR ourebia OR oribi) NOT KA (beetle OR arthropod OR oryctes OR nudivirus OR javan OR sumatran OR “one horned” OR snake OR chicken* OR human* OR Newcastle OR arabi* OR tragus OR aquaculture OR waterborne).

### Appendix B.3. Adapted for Web of Science

TS = Africa* AND TS = (virus OR viral) AND TS = (loxodonta OR “african elephant” OR giraff* OR syncerus OR “african buffalo” OR “cape buffalo” hippopotamus OR choeropsis OR rhinoceros OR ceratotherium OR diceros OR “equus zebra” OR “equus africanus” OR grevyi OR quagga OR phacochoerus OR warthog OR potamochoerus OR bushpig OR “red river hog” OR aepyceros OR impala OR alcelaphus OR hartebees* OR connochaetes OR wildebees* OR damaliscus OR tsessebe OR bonteb* OR blesb* OR antidorcas OR springb* OR raphicerus OR steenb* OR grysbok OR tragelaphus OR kudu OR koedoe OR nyala OR bongo OR bushbuck OR bosbok OR sitatunga OR taurotragus OR eland OR hippotragus OR sable OR roan OR oryx OR gemsb* OR pelea OR rheb* OR redunca OR reedbuck OR rietbok OR “kobus ellipsiprymnus” OR waterb* OR “kobus leche” OR lechwe OR “kobus kob” OR “kobus vardonii” OR puku OR cephalophus OR sylvicapra OR philantomba OR duiker OR oreotragus OR klipspringer OR spekei OR leptoceros OR “Gazella dorcas” OR eudorcas OR nanger OR addax OR “capra nubiana” OR “nubian ibex” OR beatragus OR hirola OR ammotragus OR “barbary sheep” OR dorcatragus OR madoqua OR “dik-dik” OR okapia OR okapi OR neotragus OR “royal antelope” OR suni OR litocranius OR gerenuk OR hyemoschus OR chevrotain OR ourebia OR oribi) NOT TS = (beetle OR arthropod OR oryctes OR nudivirus OR javan OR sumatran OR “one horned” OR snake OR chicken* OR human* OR Newcastle OR arabian OR tragus OR aquaculture OR waterborne) AND TI = Africa* AND TI = (virus OR viral) AND TI=(loxodonta OR “african elephant” OR giraff* OR syncerus OR “african buffalo” OR “cape buffalo” hippopotamus OR choeropsis OR rhinoceros OR ceratotherium OR diceros OR “equus zebra” OR “equus africanus” OR grevyi OR quagga OR phacochoerus OR warthog OR potamochoerus OR bushpig OR “red river hog” OR aepyceros OR impala OR alcelaphus OR hartebees* OR connochaetes OR wildebees* OR damaliscus OR tsessebe OR bonteb* OR blesb* OR antidorcas OR springb* OR raphicerus OR steenb* OR grysbok OR tragelaphus OR kudu OR koedoe OR nyala OR bongo OR bushbuck OR bosbok OR sitatunga OR taurotragus OR eland OR hippotragus OR sable OR roan OR oryx OR gemsb* OR pelea OR rheb* OR redunca OR reedbuck OR rietbok OR “kobus ellipsiprymnus” OR waterb* OR “kobus leche” OR lechwe OR “kobus kob” OR “kobus vardonii” OR puku OR cephalophus OR sylvicapra OR philantomba OR duiker OR oreotragus OR klipspringer OR spekei OR leptoceros OR “Gazella dorcas” OR eudorcas OR nanger OR addax OR “capra nubiana” OR “nubian ibex” OR beatragus OR hirola OR ammotragus OR “barbary sheep” OR dorcatragus OR madoqua OR “dik-dik” OR okapia OR okapi OR neotragus OR “royal antelope” OR suni OR litocranius OR gerenuk OR hyemoschus OR chevrotain OR ourebia OR oribi) NOT TI = (beetle OR arthropod OR oryctes OR nudivirus OR javan OR sumatran OR “one horned” OR snake OR chicken* OR human* OR Newcastle OR arabian OR tragus OR aquaculture OR waterborne).
